# Supplementary figures and images for: On the Therapeutic Potential of ERK4 in Triple-Negative Breast Cancer
Source: Cancers (Basel). 2022 Dec 21;15(1):25. doi: 10.3390/cancers15010025 (PMC9817496; doi:10.3390/cancers15010025)

1C

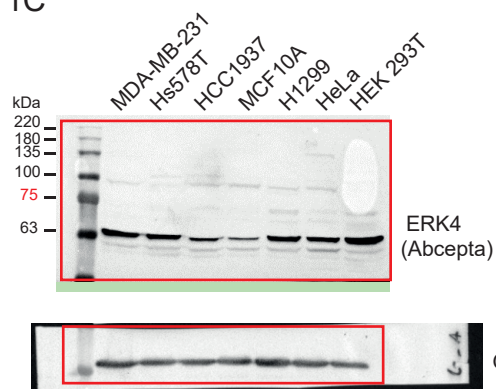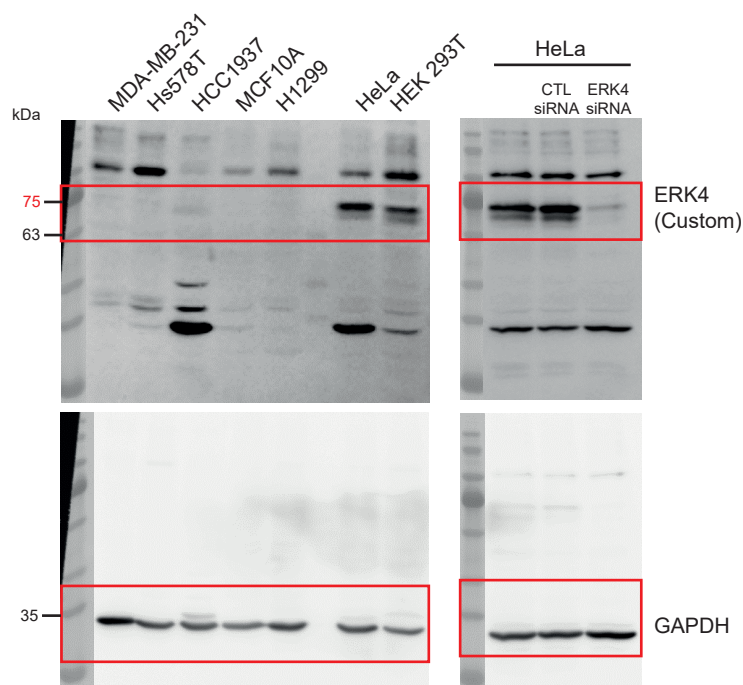

1E

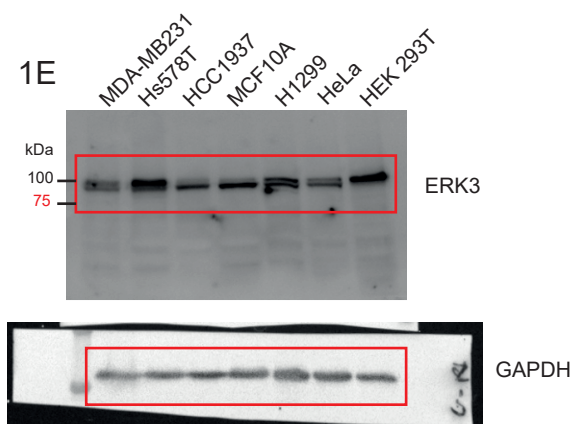

1D

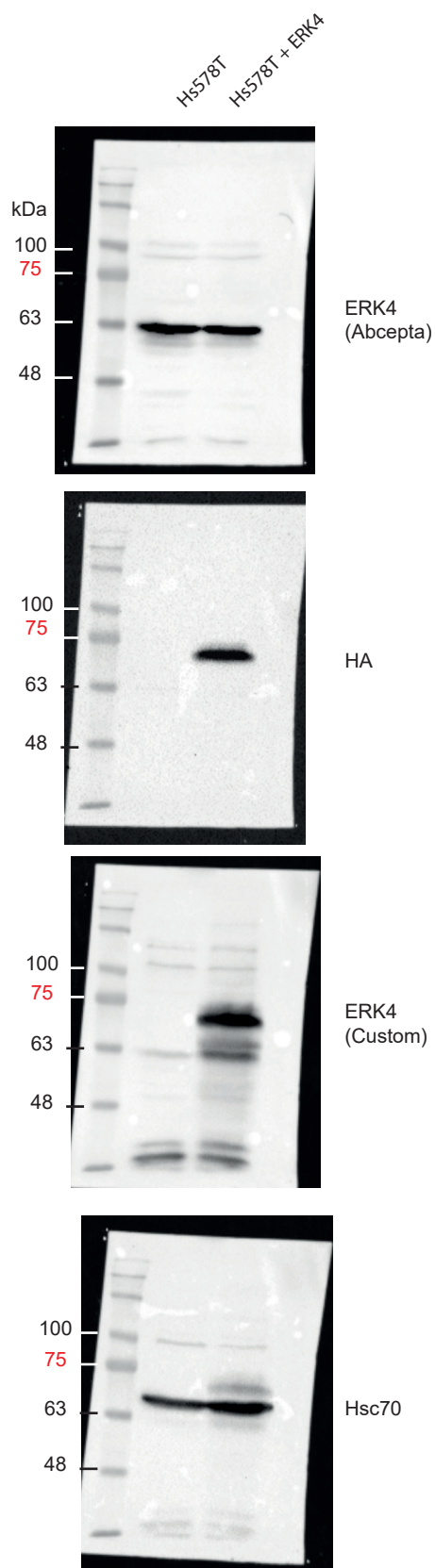

2B

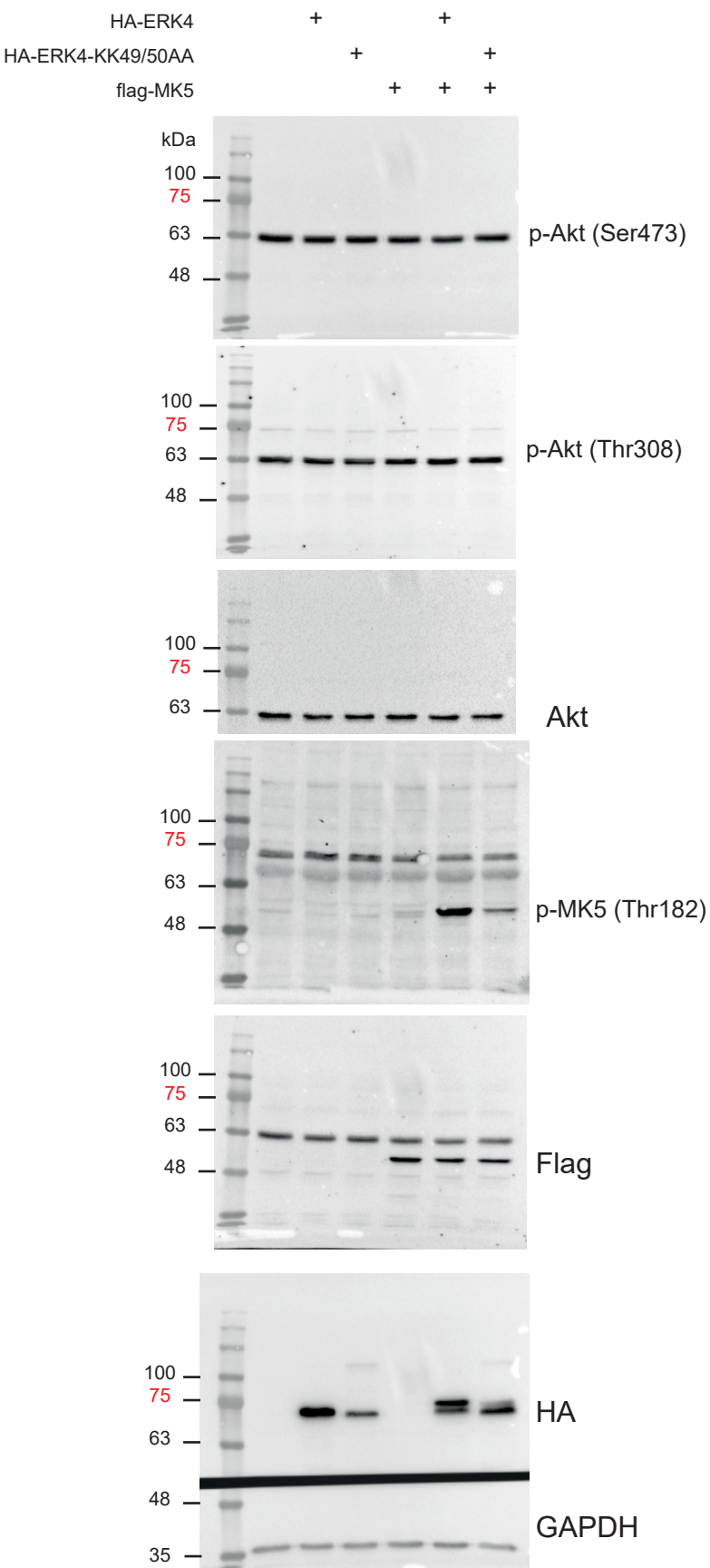

2C

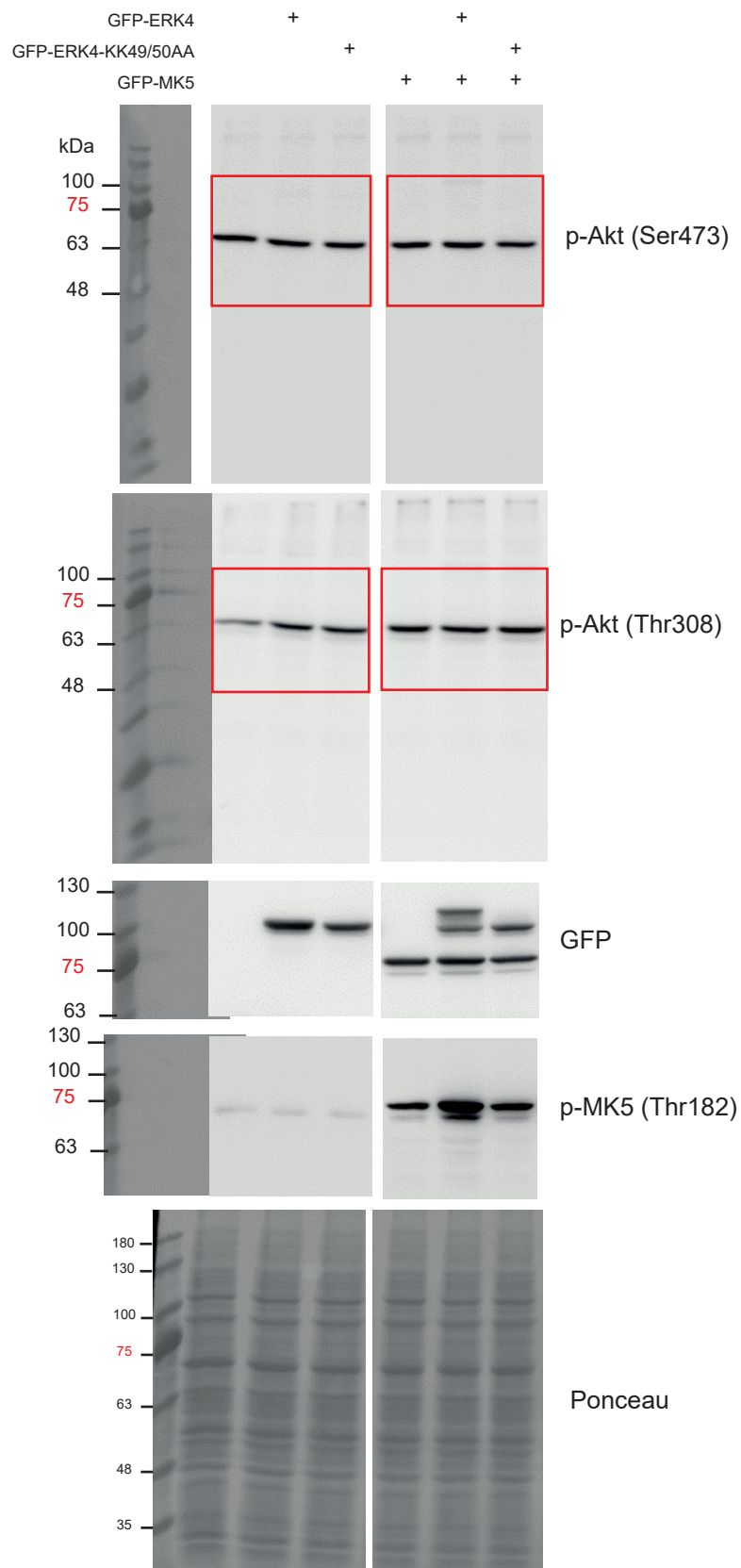

Supplement: Supplementary file 1 [file cancers-15-00025-s001.zip › cancers-2029799-supplementary.pdf]
